# Orally Administered Drugs and Their Complicated Relationship with Our Gastrointestinal Tract

**DOI:** 10.3390/microorganisms12020242

**Published:** 2024-01-24

**Authors:** Stavros Bashiardes, Christina Christodoulou

**Affiliations:** Molecular Virology Department, Cyprus Institute of Neurology and Genetics, Iroon Avenue 6, Nicosia 2371, Cyprus; cchristo@cing.ac.cy

**Keywords:** oral administration, drugs, bioavailability, microbiota, treatment efficacy, gastrointestinal tract, genetic background

## Abstract

Orally administered compounds represent the great majority of all pharmaceutical compounds produced for human use and are the most popular among patients since they are practical and easy to self-administer. Following ingestion, orally administered drugs begin a “perilous” journey down the gastrointestinal tract and their bioavailability is modulated by numerous factors. The gastrointestinal (GI) tract anatomy can modulate drug bioavailability and accounts for interpatient drug response heterogeneity. Furthermore, host genetics is a contributor to drug bioavailability modulation. Importantly, a component of the GI tract that has been gaining notoriety with regard to drug treatment interactions is the gut microbiota, which shares a two-way interaction with pharmaceutical compounds in that they can be influenced by and are able to influence administered drugs. Overall, orally administered drugs are a patient-friendly treatment option. However, during their journey down the GI tract, there are numerous host factors that can modulate drug bioavailability in a patient-specific manner.

## 1. Introduction

There are numerous modes of drug delivery, and each one has its advantages and disadvantages. The main modes of delivery include (but are not confined to) intravenous, intramuscular, and oral administration [1]. Intravenous delivery involves drug administration directly into blood circulation through a needle that pierces a blood vessel and presents the fastest route of delivery and bioavailability. Intramuscular administration relies on the high concentration of vasculature in the injected muscle for drug absorption. Although effective modes of administration, intravenous and intramuscular modes both necessitate the unpleasantries and pain of needle use [2].

Oral administration is a far more patient-friendly route with practical as well as psychological benefits. The vast majority of pharmaceutical compounds currently available on the market that are intended for human use are orally administered. In fact, these orally administered compounds account for up to 90% of total pharmaceutical compounds intended for human use [3]. Orally administered drugs represent the generally preferred delivery route for both patients and treating physicians since they are convenient, non-invasive, and importantly allow for self-administration, all of which increase patient compliance.

**Figure 1:**
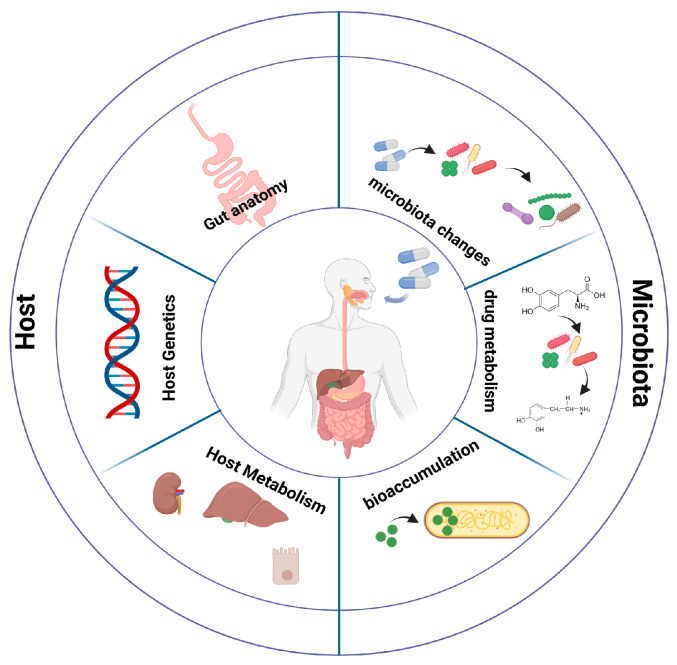
Factors affecting drug bioavailability. Host factors modulating drug bioavailability include gut anatomy, host genetics, and host metabolism. Interactions between drugs and microbiota can result in composition (and function) alterations. Furthermore, drug metabolism by bacterial components of the microbiota community and bioaccumulation of drugs in the intestinal lumen can lead to drug bioavailability modulation.

Drugs administered orally will generally be intended for localized action within the gastrointestinal (GI) tract, or for passage through the GI tract lining and into systemic circulation for transfer to the relevant location so that their activity can be exerted. Although oral administration is a preferred mode of drug administration, pharmacologically, the GI tract represents a challenging and complex environment [4]. These complexities are brought about by numerous factors including GI tract physiology as well as GI tract environment (Figure 1).

Beyond the physiology of the GI tract, the trillions of microorganisms colonizing our gut have emerged as a metabolic powerhouse capable of chemically altering xenobiotics presented to them [5]. The bacterial metabolism of xenobiotics can have a dramatic impact on drug bioavailability and treatment efficacy. Conversely, xenobiotics can impact the bacterial populations colonizing the gut with potential downstream implications. In the context of drug bioavailability, our genetic background presents an additional layer of complexity impacting treatment efficacy (Figure 1).

In this review, we discuss the intricate relationship between ingested xenobiotics and the host being treated with these compounds.

## 2. Gastrointestinal Tract

The GI tract can be thought of as a muscular tube, from mouth to anus, involved in the digestion of foods that we ingest, and the subsequent absorption of nutrients from these digested foods, while also playing a role in excreting waste products [6]. Although this oversimplified understanding of the GI tract holds truth, in reality, the GI tract is a highly complex environment presenting many challenges for drug delivery (Figure 1). If we classify the GI tract into upper and lower GI tracts, the former includes the stomach and duodenum, while the latter includes the jejunum and ileum, followed by the large intestine [7]. Although drugs can be formulated to enhance absorption in the upper or lower GI tract, the highest degree of absorption takes place in the small intestine, assisted by its large surface area [8] that has recently been estimated to be approximately 32 m^2^ [9] (a deviation from the traditionally stated 200 m^2^, but still a substantial surface area). A topic of intense research and development is that of drug delivery systems to allow for controlled delivery of oral drugs, including hydrogels and nanoparticles (among others) [10,11,12]. For ingested drugs to enter systemic circulation (and travel to the location where they will exert their effect), they must first cross the gut mucosa, and upon absorption enter circulation. A highly structured epithelial cell barrier in the gut lumen creates a controlled passage for components from the gut luminal content through to the underlying gut tissue and vasculature. There are two main routes for drug passage through the gut mucosa, namely by paracellular diffusion (passive movement through the tight junctions between epithelial cells), and via the transcellular active transport through the cell by an endocytosis-mediated route, which constitutes the main route for drug uptake [13]. Transcellular transport is directly affected by the lipid solubility of a drug, highlighting the importance of pharmaceutical synthesis and delivery methods of orally administered drugs [14].

The road to drug GI tract absorption is “paved” with numerous challenges. Numerous factors relating to GI tract physiology can modulate drug bioavailability and can be responsible for the interindividual heterogeneity inherent in drug treatments. A lengthy discussion and details of the GI tract variability that can have an effect on drug absorption and pharmacokinetics of these drugs go beyond the scope of this review and have been discussed nicely elsewhere [4,15]. To put the issue in context, however, we shall briefly mention and touch upon some of these physiological aspects of the GI tract impacting drug bioavailability. The length of the GI tract (and components of it such as the small intestine and colon) shows significant interindividual variability indicated by the broad range in average small intestine length determined between 298–1030 cm, while the average colon length ranges from 80–313 cm [16]. The time taken to pass through specific locations of the GI tract, the transit time, can affect drug dosage, particularly for drugs that are absorbed in specific regions or have region-specific actions. Transit time through the small intestine can range from 2–6 h between individuals, while transit time through the colon can vary between 6–70 h [6].

Mucosal surfaces have a protective mucus lining. Mucus represents an important component of the GI architecture, acting as a protective layer to epithelial cells keeping pathogens at bay and preventing mechanical stress. It is composed of the mucin protein (and proteoglycans) that can present a challenge for drug delivery and absorption. The stomach has a double layer of mucus protecting the stomach lining from gastric acid. In the small intestine, there is a single mucus layer, whereas the colon has a double layer of mucus [17]. The pH varies along the GI tract from stomach to rectum, with interindividual variability being apparent due to food, age of individuals, as well as circadian rhythmicity [18], and can have implications on design and drug dosage [19].

## 3. Host Factors Affecting Interindividual Drug Responses

### 3.1. Genetic Background

That interindividual responses to drugs taken can vary considerably is an established fact, and is more common and less appreciated than expected. This is highlighted by the fact that a response rate of between 50–70% was determined for a wide variety of common drugs [20], a statistic that is astonishing if we consider that almost half of the patients being administered a broad range of common drugs do not see benefits to treatment. Furthermore, adverse drug reactions (ADRs) pose a health risk as well as an economic burden. In the USA and Europe, approximately 7% and 3.5%, respectively, of hospital admissions result from ADRs [21,22]. These statistics clearly make a case for developing and implementing personalized treatment approaches with the aim of enhancing drug treatment efficacy while reducing the probability of an ADR. In 2001, the first draft of the human genome was completed, a landmark event in science history, bringing numerous exciting prospects and potential challenges [23,24]. One of the numerous grand challenges and prospects following the sequencing of the human genome involved the determination of genetic contributors to human disease and also the genetic variations that modulate response to drug treatment [24]. This can be exemplified by variations in genes that encode liver enzymes that can lead to changes in the functioning of enzymes and by extension to drug metabolism [25]. Pharmacogenetics was born out of the prospects presented by knowledge of the human genome sequence, with an aim to determine variants affecting drug responses and in this way provide a predictive tailored treatment approach based on an individual’s genetic background (Figure 1). Implementing the knowledge gained on the effects of variants on drug responses in the clinic can be a gradual process requiring clear clinical guidelines. In this context, the Clinical Pharmacogenetics Implementation Consortium has published numerous guidelines relevant to a wide range of drugs and the genes they interact with [26]. Furthermore, a study conducted by the Dutch Pharmacogenetics Working Group in outpatient clinics over a 7-year period indicated that patients were exposed to pharmacogenetics drugs (drugs with identified gene interactions) as many as 51.3 million times, and astonishingly, testing a panel of three genes alone (*CYP2D6*, *SLCO1B1*, and *CYP2C19*) could predict up to 95% of pharmacogenetic drugs with individuals possessing a vulnerable genotype [27].

The host’s genetic background is clearly important in potentially determining the efficacy of drug treatments (Figure 1). There is, therefore, real validity in considering the implementation of pre-emptive and predictive genetic testing so that a more personalized and individual patient-tailored treatment approach can be followed. Even so, it has been estimated that the underlying genetic background of individuals accounts for 20–90% of observable variability in the response to a given drug treatment [28]. There are, therefore, numerous pieces of the puzzle in drug response variability between individuals.

### 3.2. Xenobiotic Metabolism

Host metabolism of xenobiotics mainly occurs in the liver with an overall goal being the excretion of either endogenous or exogenous compounds (Figure 1). Xenobiotics include pharmaceutical drugs administered for therapeutic purposes but also include additional naturally occurring compounds such as phytochemicals present in ingested foods. The metabolic processes undertaken during this activity can be sequential or can occur at the same time (or even in reverse order), with the aim to convert the relevant compounds to more polar substances that can be excreted by the liver, gut, or kidneys. The metabolic reactions that take place to facilitate the excretion of substances mainly take place in three phases, namely phase I, phase II, and phase III. Relevant drug metabolism pathways take place in the liver, gut enterocytes, and also in the kidneys [29]. During phase I, catalysis takes place predominantly by the cytochrome P450 family of genes [30], a group of enzymes that are membrane-bound and located in the endoplasmic reticulum of hepatocytes. During this phase, lipophilic drug compounds are converted to more polar and water-soluble ones through polar side groups such as, for example, -OH and -NH_2_. Reactions in this phase include oxidation (cytochrome P450) as well as reduction and hydrolysis. The ubiquitous nature of the cytochrome P450 family’s involvement in this phase renders clinical relevance to gene variations modulating enzyme activity (and thus level and extent of metabolism). In the next phase of biotransformation, phase II, resulting substances from phase I are further metabolized with the addition of endogenous hydrophilic groups. This is carried out through transferase enzymes, with UDP-glucuronosyltransferases (UGTs) representing the most common ones. These reactions occur in the liver (where the relevant enzymes are expressed), but also in the intestinal enterocytes and in the kidney (although in the kidney, glutathione S-transferase is the main conjugating enzyme). During phase III of the biotransformation process, active transportation of compounds occurs, resulting in compounds in the liver being uptaken into hepatocytes and excreted in bile, and in the kidneys, compounds being actively transported into urine for excretion. The ATP-binding cassette transporters are mainly involved in these processes along with solute carrier transporters.

Although clear-cut pathways for the metabolism of xenobiotics exist in humans, there are numerous factors that can affect these metabolic processes. Substrates, inhibitors, and inducers of the three phases of metabolism can modulate the speed and efficiency of compound biotransformation. For example, rifampin and nafcillin can act as inducers of phase I CYP3A enzymes, whereas cyclosporin A and flavonoids can act as inhibitors of UGT and SULT enzymes, respectively, of phase II pathways. Furthermore, numerous physiological factors, such as age [31], gender [32], liver disease [33], kidney disease [34], and gene polymorphisms [35], can also have a major impact on drug metabolism.

## 4. Impact of Frequently Used Drugs on the Gut Microbiota

As discussed earlier, with regard to oral drug usage, the road to drug GI tract absorption is “paved” with numerous challenges. In addition to the physiological and structural aspects of the GI tract that can influence drug bioavailability, one additional factor whose potential impact is becoming all the more apparent is that of the gut microbiota. The gut microbiota includes bacteria, viruses (eukaryotic and bacteriophages), fungi, archaea, and parasites that colonize the GI tract. The most comprehensively studied component of the gut microbiome is the bacterial component, and this is the component that we will focus on in the current review. In the gut, the bacterial component achieves a massive colonization density with the colon estimated to reach approximately 10^11^ bacteria/mL [36]. It is estimated that over 1000 species of bacteria colonize the human gut, all of which belong to a limited number of phyla, the most dominant being Bacteroidetes and Firmicutes, with other phyla such as Actinobacteria, Tenericutes, Proteobacteria, and Verrucomicrobia being less prominent [37]. Even though these are the general compositional traits, the actual populations of bacterial species colonizing individual humans are radically different with varying concentrations of taxa [38]. Interestingly, the genomes of our microbiome were reported to contain approximately 3.3 million genes, a number that greatly outnumbers the 23,000 genes of the human genome [39]. A recent study presenting metagenomics data from 1267 gut metagenomes that included European, American, and Chinese samples reported approximately 9.8 million genes [5]. In this study, approximately 750,000 non-redundant genes were identified per sample analyzed, and of these, less than approximately 300,000 were common between more than 50% of participants whose samples were analyzed [5]. These data highlight the fact that there is an extraordinarily high number of non-redundant genes represented in each individual’s metagenome (in terms of taxonomic colonization), and also the interindividual variability that exists on the metagenome gene level. That the metagenome gene count greatly overshadows human host gene counts is indicative of the enormous metabolic potential harbored by the microbial components of our microbiota. Furthermore, interindividual variability identified at the metagenomic individual gene level is further indicative of the microbiota interindividual metabolic potential variability.

**Figure 2:**
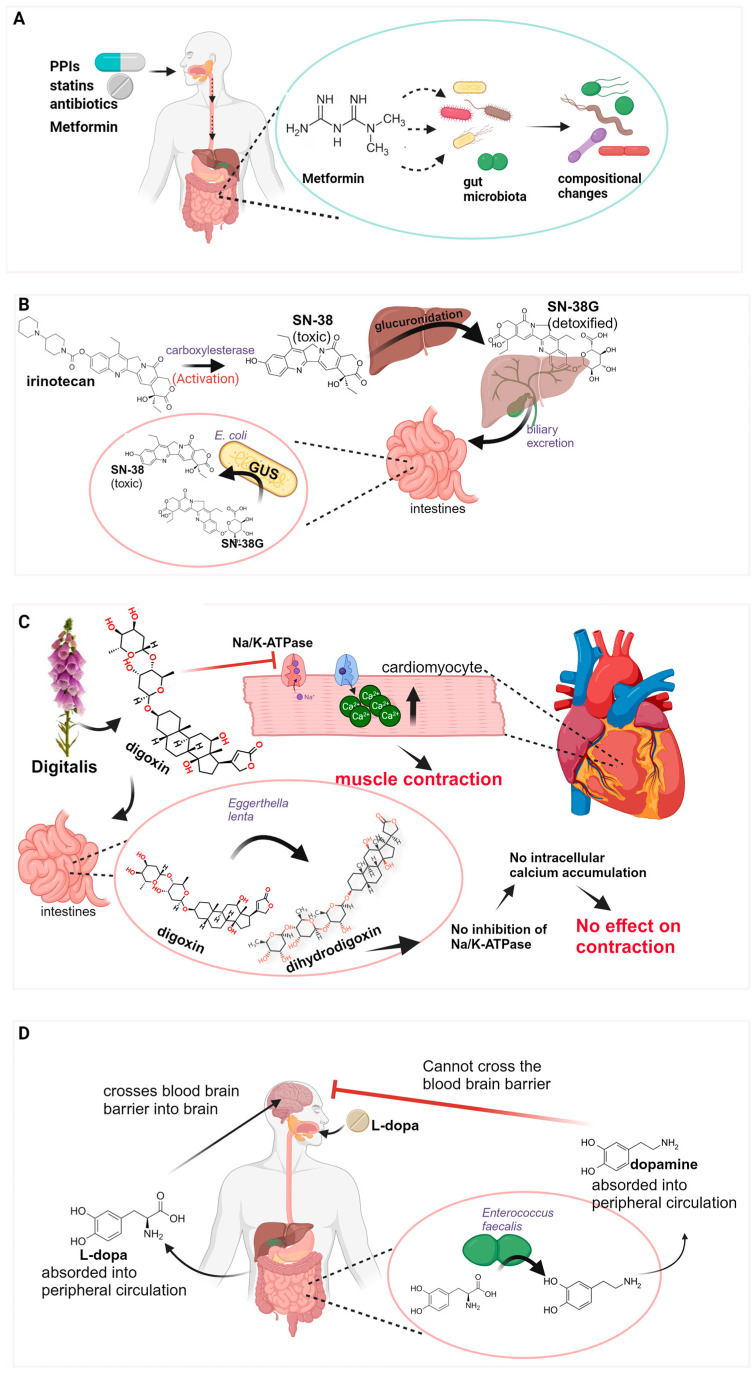
Interactions between drugs and microbiota. (**A**) Drugs leading to compositional alterations in microbiota. Metformin has been used as an example to illustrate this point. (**B**) Irinotecan activation via carboxylesterase to SN-38, the active and toxic molecule that is subsequently detoxified to SN-38G via glucuronidation in the liver and excreted in the gut lumen. *E. coli* bacterial strains using GUS enzymatic activity convert SN-38G back to the toxic SN-38 molecule. (**C**) Digoxin acts by inhibiting Na/K-ATPase pumps in cardiomyocytes, resulting in an increase in Ca^2+^ ions and more forceful contraction. Strains of *Egerthella lenta* metabolize digoxin to the inactive dihydrodigoxin, resulting in lower drug efficacy. (**D**) L-dopa is absorbed in the gut into the peripheral circulation, crosses the blood–brain barrier, and enters the brain where it is converted to dopamine. Bacterial strains in the gut metabolize L-dopa into dopamine that enters peripheral circulation and cannot cross the blood–brain barrier.

The use of prescription drugs is ubiquitous and common. In the USA, according to data from the Center for Disease Control (CDC), in a period of 30 days, at least one prescription drug has been taken by 48.6% of the population [40]. Suffice it to say that there are enormous quantities of prescription drugs being taken by the general public. With these quantities consumed, it is relevant, if not obligatory, to comprehend the potential interactions of these xenobiotics and the trillions of microorganisms that reside within our body and can be affected by or can affect (Figure 1 and Figure 2).

### 4.1. Antibiotics

Obvious culprits with a direct effect on the microbiota are the various groups of antibiotics, each with the range of bacteria that they target [41]. Use (and abuse) of antibiotics over the past few decades has been the subject of many studies [42,43], with recent studies characterizing dysbiosis caused by antibiotics, as well as the short- and long-term effects on human pathophysiology (Figure 2A). Short-term effects include a reduction in microbiota diversity as well as non-specific reduction/eradication of bacteria that may be beneficial [44], which, in turn, can allow the flourishing of pathogens. A classic example is that of *Clostridium difficile*, which can flourish following antibiotic use, leading to symptoms that range from mild to potentially lethal [45]. Recent studies are unraveling the long-term effects of antibiotic use on chronic pathologies, such as obesity [46,47], inflammatory bowel disease [48], and cardiometabolic events [49], among others.

### 4.2. Protein Pump Inhibitors

Protein pump inhibitors (PPIs) are drugs that act to limit the production of stomach acid and are commonly prescribed to treat gastroesophageal reflux as well as peptic ulcers [50]. As they have been deemed low-risk, they are widely used, being among the ten most frequently used drugs worldwide [51]. Studies show that the use of PPIs can result in an increase in enteric infections [52], and have been found to result in significant changes in microbiota composition [53] (Figure 2A). One theory is that reduced stomach acid brought about by PPI use removes the “acid barrier” between the upper and lower GI tract, allowing oral bacteria to colonize the lower gut [53]. In this context, a dangerous precedent of PPI use is the associated increase in *Clostridium difficile* infections [54], which warrants careful consideration and a potential re-evaluation of criteria for PPI prescription or at the very least careful observation during or following use, particularly long-term use.

### 4.3. Statin Drugs

Statins are drugs used in the management of dyslipidemia and to reduce the risks of cardiovascular disease development [55]. They inhibit HMG-CoA reductase, a rate-controlling enzyme in the pathway leading to cholesterol synthesis in the liver. This results in the increased expression of the LDL receptor on hepatocytes, and consequently increased clearance of LDL and VLDL molecules that, in turn, results in a reduction in blood cholesterol and triglycerides [56]. Their use is highly prevalent with over 200 million individuals using them worldwide. Relevantly, the prevalence of obesity is alarming with over 1.9 billion people worldwide, and with co-morbidities including cardiovascular disease and dyslipidemia [57]. One of the hallmarks of obesity is chronic low-grade inflammation [58], with evidence pointing toward a microbiota component in inflammation development [59]. A recent study addressed the issue of identifying marker microbiota profiles for obesity and associated systemic inflammation [60]. The Bacteroides 2 enterotype (Bact2), characterized by enrichment in *Bacteroides* genera, was found to be associated with obesity and correlated to an increase in body mass index [60] where a higher prevalence of the Bact2 enterotype was found. This enterotype has also been associated with a higher plasma C-reactive protein concentration, considered a hallmark of inflammation [61]. It is worth noting that apart from obesity, the Bact2 enterotype has been associated with various other states of inflammation, such as Crohn’s disease, among others [62]. Interestingly, obese individuals taking statins have been found to have a lower prevalence of the Bact2 enterotype compared with obese individuals not taking statins [63] (Figure 2A). This raises the possibility that the use of statins in obese individuals can result in the deviation from a pathogenic Bact2 enterotype to a less pathogenic (or at least less inflammatory associated) microbiota composition. Such observations represent an interesting notion and warrant further investigation through large clinical studies to address the issue of confounding by indication and also to determine uniformity in observations across ethnic groups.

### 4.4. Metformin

Metformin, a commonly prescribed drug for type 2 diabetes, was the first drug other than antibiotics shown to result in microbiome treatment signatures (Figure 2A) and highlighted the need for treatment stratification in the interpretation of microbiome data [63].

The currently predominating theory on the mechanisms leading to drug-imposed gut microbiome alterations suggests that alterations in gut microenvironments brought on by drugs ultimately lead to bacterial growth modulation. This effect on bacterial growth can either lead to the enhancement of specific strains or, conversely, growth inhibition of specific strains. The gut microbiome’s importance in the clinical effects of metformin is indicated by the fact that intravenous administration of the drug brings about a limited effect on reducing plasma glucose concentrations [64]. Furthermore, fecal transplantation from human donors that were administered metformin into germ-free mice on a high-fat diet resulted in an increased glucose tolerance [65]. Although numerous mechanisms have been postulated, the exact mechanism through which the administration of metformin, and the resulting microbiome changes, leads to an increased clinical effect in regulating blood glucose levels still remains unclear. One effect can be to bring about microbiome compositional changes that alleviate the dysbiotic signatures identified in type 2 diabetes patients [66]. Reproducible compositional changes, identified across studies, include elevated levels of Escherichia and lower levels of Intestinibacter when comparing individuals treated with metformin to untreated individuals [65,67]. The changes in abundance of these bacteria were postulated to be involved in the enhanced effects of metformin through the consequential increase in short-chain fatty acids (SCFAs) that are produced in the GI tract [65]. The beneficial role of SCFAs on glucose homeostasis through intestinal gluconeogenesis regulation has been described in rodents [68]. The proposed mechanisms resulting from metformin administration highlight the complexity of drug–microbiome interactions. Metformin has been associated with the release of gut hormones by enteroendocrine cells, an event triggered by SCFSs. Therefore, the increase in SCFA-producing bacteria brought about by metformin can indirectly lead to increased secretion of these gut hormones [69] and ultimately induce the effects of metformin.

The list does not end here as there are numerous additional examples of identifiable microbiome alterations brought on by drug use, such as in the case of laxatives, opioids, and paracetamol [70]. In fact, a large-scale study investigating the effects of more than 1000 drugs on a group of 40 bacterial strains astonishingly revealed that 24% of tested drugs impacted the growth of one or more strains included in the assay [71]. The impact of administered drugs on various aspects of the microorganisms colonizing our gut is profound and far-reaching.

## 5. Bacterial Drug Metabolism

A recent estimate of the non-redundant microbiota gene content of oral and gut microbiota from 3655 samples revealed enormous genetic heterogeneity and approximately 23.9 million non-redundant genes in oral samples and 22.2 million non-redundant genes in gut samples [72]. This enormous and diverse array of genes provides the microbiota commensals with a massive metabolic potential with regard to metabolizing ingested compounds, including drugs, to varying capacities and complexities. This lesson was unfortunately learned the harsh way in 1993 following the death of 16 oncology patients after they were co-administering the antiviral sorivudine and the anticancer drug 5-fluorouracil [73], an outcome credited to components of the relevant patient gut microbiota. Sorivudine (1-β-d-arabinofuranosyl-E-(2-bromovinyl)uracil), a synthetic thymidine analog, has been shown to represent an effective antiviral drug against a number of herpesviruses such as Epstein–Barr virus and herpes simplex 1 virus [74]. Conversely, 5-fluorouracil (5-FU) represents a staple of treatment strategies for numerous solid tumors [75]. Each drug represents an effective treatment strategy for its respective target in its own right; however, coadministration can result in outcomes ranging from undesirable to catastrophic [73]. The main culprits of such outcomes are components of the intestinal microbiota possessing the metabolic capacity to hydrolyze sorivudine to bromovinyluracil (BVU). Examples of bacteria capable of carrying out such activity include *Bacteroides fragilis*, *Bacteroides eggerthii*, and *Bacteroides vulgatus* [76]; these bacteria lead to the inactivation of hepatic dihydropyrimidine dehydrogenase that detoxifies 5-FU. Bacterial enzymatic activity results in increased 5-FU abundance systemically and, consequently, an increase in toxicity.

### 5.1. Bacterial β-Glucuronidase and Drug Toxicity

Irinotecan, a potent topoisomerase I inhibitor, limits rapid cell proliferation in tumors and is a commonly used treatment agent for solid tumors. Although effective, irinotecan treatment results in late-onset diarrhea with approximately 30% of patients experiencing acute diarrhea [77]. Irinotecan is a prodrug metabolized into the active metabolite SN-38 through the activity of carboxylesterases following intravenous administration [78]. It is subsequently transported to the liver where the addition of glucuronic acid takes place, and through this process, SN-38 is detoxified to SN-38G. The resulting SN-38G is excreted into the GI tract [79] where SN-38G is subjected to the metabolic potential of commensal strains with β-glucuronidase (GUS) enzymatic activity that results in glucuronic acid removal and consequent reactivated SN-38. This results in damage to the GI tract and diarrhea that is dose-limiting (Figure 2B).

Non-steroid anti-inflammatory drugs (NSAIDs) are a class of drugs used for pain relief, as anti-inflammatory drugs, and for their antipyretic properties. Their popularity is reflected in the fact that over 30 million people take NSAIDs daily [80]. Apart from their undoubtable benefits, there are toxicity-related side effects that are most prominently displayed in gastrointestinal complications leading to over 1 million hospitalizations annually [80]. As in the case of irinotecan where the active metabolite, SN-38, is inactivated through the addition of glucuronic acid, NSAIDs are also inactivated through glucuronidation in the liver and excreted into the gut lumen. As with irinotecan, NSAIDs are also subject to bacterial-mediated β-glucuronidase reactivation resulting in dose-limiting toxicities [81]. Furthermore, reactivated compounds through the action of bacterial GUS enzyme activity can be reabsorbed into circulation and go through subsequent enterohepatic recirculation cycles [82]. GUS enzymatic activity is derived from the gusA gene, a component of the GUS operon identified and studied extensively in *E. coli* [83]. Interestingly, a comprehensive characterization of GUS enzymes in the human microbiome identified 279 unique proteins, and taxonomically the bacterial strains that encode these GUS enzymes go beyond the Enterobacteriaceae family, where it has been established that members possess a GUS operon [84,85].

Given the nature of elevated toxicity relating to irinotecan or/and NSAIDs due to microbiota GUS enzymatic activity, an obvious target to limit toxicity and increase drug efficacy is the enzyme itself. Inhibitors that target β-glucuronidase enzymatic activity have been identified and tested, initially by Fittkau et al. [86], showing reduced toxicity and gastrointestinal damage in mice treated with irinotecan. Additional (and more efficient) inhibitors targeting bacterial β-glucuronidase enzymatic activity were later described [81]; these inhibitors targeted a structural loop region specific to bacterial β-glucuronidase that is not present in mammalian β-glucuronidase, resulting in bacterial-targeted inhibitors that do not affect mammalian enzymatic activity. The use of a GUS inhibitor in preclinical mouse cancer models indicated the potential of such targeting strategies as there was an overall increase in chemotherapeutic efficacy, with simultaneous improvement in gut damage and survival [87]. This raises the interesting possibility of inhibitor use in patients treated with irinotecan.

### 5.2. Digoxin

*Digitalis purpurea* (commonly known as foxglove) is a plant used for centuries for its medicinal properties [88]. Although digitalis leaves themselves are poisonous to humans [89], pure isolated compounds such as digoxin, a cardiac glycoside, can be administered at low doses to patients with heart failure and atrial fibrillation [90,91]. Digoxin functions by inhibiting Na/K-ATPase pumps in cardiomyocytes, ultimately leading to an increase in intracellular calcium ion abundance, resulting in a more forceful contraction. The use of digoxin is still prevalent, with users in the USA at 402 per 100,000 during the period 2016–2017 [92].

The anaerobic Coriobacteriia *Eggerthella lenta* is a commonly found bacteria in the GI tract of humans that has been known for decades to be involved in the metabolism of digoxin into an inactive metabolite, dihydrodigoxin, and by doing so modulating drug efficacy (Figure 2C). Recent studies have identified a cytochrome-encoding operon, the *cgr* operon, encoding Cgr2 (cardiac glycoside reductase 2), responsible for digoxin metabolism [93]. The prevalence of *Eggerthella lenta* was found to be 81.6% in individuals tested, with the prevalence of the *cgr2* gene being 74.7% [93]. Cgr2 enzymatic activity can metabolize toxic compounds of foxgloves, including digoxin. This metabolic activity does not seem to confer any benefit to the bacteria itself but rather seems to confer a benefit to the host through protection against the ingestion of plant toxins [93]. Early attempts at understanding the growth dynamics of *Eggerthella lenta* indicated that its growth required the presence of the amino acid arginine [94]. Later studies confirmed the enhancement of growth in the presence of arginine, and interestingly also showed the concurrent inhibition of digoxin metabolism to an inactive form [95]. Experiments with the use of *Eggerthella lenta* monocolonized germ-free (GF) mice showed that animals consuming a diet consisting of increased dietary protein, led to an elevation in digoxin in serum compared with animals consuming a diet lacking protein [96]. This raises the obvious question of whether a targeted dietary intervention can effectively be implemented to limit *Eggerthella lenta* metabolism of digoxin in patients with high levels of metabolizing bacterial strains. Furthermore, a mechanistic understanding of arginine cgr operon inhibition could potentially pave the way for inhibitor-based interventions as in the case of GUS inhibitor use in cancer therapy (Section 5.1).

### 5.3. Levodopa

Parkinson’s disease pathology displayed by patients is characterized by the loss of dopaminergic neurons through neurodegeneration, and replacement therapy through levodopa (L-dopa) administration represents the most prevalent treatment strategy since the 1960s [97]. Levodopa is administered orally, and following oral ingestion, it passes through the stomach and reaches the duodenum and jejunum where absorption takes place. Although L-dopa is an effective treatment strategy for patients, astonishingly, only 1–5% of administered L-dopa reaches the central nervous system [98]. Decarboxylation of L-dopa in peripheral tissue leads to a reduction in bioavailability and patients are, therefore, routinely co-administered L-dopa with decarboxylase inhibitors (such as carbidopa) to reduce peripheral decarboxylation [99]. Adding to the complexity surrounding L-dopa bioavailability, components of the microbiome have emerged as modulators of bioavailability and treatment efficacy. Since the early 1970s, it has been known that the bacteria colonizing Parkinson’s patients’ guts metabolize levodopa, although mechanistic determination and specific bacterial strains involved were not determined until recently. Current mechanisms through which bacteria act to modulate bioavailability include direct L-dopa metabolism and L-dopa absorption modification [100,101]. Recent studies have identified the bacterial strains *Enterococcus faecalis* and *Enterococcus faecium* as metabolizers of L-dopa through the tyrosine decarboxylase gene (*TyrDC*) that they possess [100,101]. Metabolism of L-dopa in the GI tract by these bacterial enzymes results in the peripheral conversion to dopamine that cannot cross the blood–brain barrier, rendering the bioavailable drug dose diminished (Figure 2D). Importantly, decarboxylase inhibitors co-administered with L-dopa to Parkinson’s patients were found to be ineffective in their ability to inhibit bacterial decarboxylase activity in the gut [100]. A tyrosine mimic, (S)-α-fluoromethyltyrosine, was identified and utilized in a mouse preclinical model demonstrating inhibition of bacterial tyrosine decarboxylase activity and the resulting increase in serum L-dopa levels [100]. The use of the tyrosine mimic represents an interesting approach to limiting the activity of bacterial tyrosine decarboxylases in a preclinical model and certainly warrants additional investigation as to its applicability and efficiency for human use.

Although not a focus of this review, it is important to note that in addition to pharmaceutical compounds, numerous ingested naturally occurring compounds in foods we eat (such as phytochemicals) interact in a complex manner with the microbiota community resulting in far-reaching physiological effects through antioxidant and anti-inflammatory (among others) pathways [102,103]. This important aspect of xenobiotic metabolism by the gut microbiota has been reviewed elsewhere [102,103,104].

### 5.4. The Scale of Drug Biotransformation in the Gut

In the context of drug efficacy modulation through microbial metabolic activity, the extent to which this occurs in orally administered drugs is an important and relevant question to answer. Zimmermann et al. [105] addressed this question by conducting a large-scale study to determine how prevalent the ability to metabolize commonly administered drugs was among representative bacteria colonizing the human gut. In this study, the ability of 76 representative bacteria colonizing the human gut to metabolize 271 orally administered drugs was tested [105]. Interestingly, out of the 271 drugs tested, 176 (66%) were found to be metabolized by at least one of the bacteria tested, highlighting the enormous drug-metabolizing capacity of the bacteria that colonize our gut. Indicative of the fact that bacteria have different metabolizing capacities, was the finding that two of the bacteria tested were able to metabolize the majority of the 176 drugs tested. Specifically, *Bacteroides dorei* was able to metabolize 164 drugs, while another bacterium, *Clostridium* sp., was able to metabolize 154 drugs [105].

## 6. Bioaccumulation

Our understanding of bacteria–drug interaction modes and complexities was further expanded by a recent study analyzing the impact of bioaccumulation and its ramifications [106]. The process of bioaccumulation involves the intracellular storage and accumulation of a drug without its chemical modification and, as a consequence, results in reduced bioavailability (Figure 1). Bioaccumulation is not a new concept and has commonly been addressed in the context of bioremediation of heavy metal-polluted environmental sites [107]. The ability of 25 representative bacterial strains that colonize the human gut to bioaccumulate 15 structurally diverse drugs was investigated by Klünemann et al. [106]. They showed that 17 novel bacteria–drug interactions identified in this study take place via bioaccumulation events. To understand further the underlying processes involved, the antidepressant duloxetine was used in the study as a sample drug with a predefined bacterial community. This revealed very interesting findings in that the effects of drug bioaccumulation appear to go beyond just reduced drug bioavailability but can also have far-reaching bacterial community effects. Klünemann et al. found that bioaccumulation of a drug can change metabolite secretions from the bioaccumulating strain, and these changed secretions can affect the growth of other bacteria in the community [106]. Furthermore, in addition to bacterial community effects, the direct impact bioaccumulation can have on the host was shown using *Caenorhabditis elegans* as an in vivo model system. Under normal circumstances, duloxetine is a movement inhibitor in *Caenorhabditis elegans*; however, in the presence of bioaccumulating bacteria, movement inhibition of duloxetine was attenuated.

## 7. Microbial Diet Molecule Modification

Foods and drugs that we consume are supplemented with numerous excipients, components included in pharmaceutical/food products, that serve a specific purpose and are considered inert. These excipients can constitute a substantial proportion of a drug’s formulation, in some cases being as high as 90%, and play a role in important aspects of drug characteristics such as stability [108]. As a result of orally ingested drugs, excipients occupy the luminal content of the gut. The extent to which these compounds can have an effect on drug bioavailability has been unclear. A study conducted by Zou et al. [109] determined the impact of excipients on drug absorption by the gut. In this study, 136 commonly utilized excipients in the pharmaceutical industry were screened to assess their potential impact on drug absorption through the intestine. The study focused on the OATP2B1 transporter, which is involved in the cellular uptake of a broad spectrum of drugs that include the antihistamine fexofenadine [110]. Of the 136 excipients screened, 24 were identified as inhibiting drug transport. These included a common dye exponent utilized in the pharmaceutical and food industry, FD&C Red No. 40, which was identified as an inhibitor of OATP2B1. Mouse studies revealed that FD&C Red No. 40 efficiently inhibited OATP2B1 intestinal absorption of fexofenadine, resulting in its reduced identified abundance in serum. Interestingly, with the use of ex vivo experiments, Zou et al. [109] revealed that microbiome samples from healthy donors, as well as individual human gut microbial isolates, were able to metabolize FD&C Red No. 40 (as well as other azo dyes), thus generating metabolites that were not inhibitory toward OATP2B1 activity. This presents an interesting notion whereby bacterial components of the microbiome are able to rescue the negative impacts commonly used exponents have on drug absorption in the gut.

## 8. Predicting Gut Microbiota–Xenobiotic Interactions

The capacity of gut microbiota components to interact with xenobiotics, and through this interaction result in biotransformation of these compounds, is now well established. Conversely, the capacity of xenobiotics to cause compositional changes in the microbiota community structure has also been established. The clinical relevance of comprehensively understanding microbiota–drug interactions is becoming all the more important, as is the relevance of incorporating this understanding into clinical practice. The power to predict such interactions could limit the extent of microbiota compositional changes induced by drugs (resulting in dysbiotic events) as well as adverse effects of microbial xenobiotic biotransformation and ultimately has the potential to reshape patient oral drug prescription. The power to make these predictions, however, represents a daunting task due to its immense complexity. As research relating to drug–microbiota interactions has led to the generation and accumulation of relevant data, our understanding of individual patient responses becomes more insightful. These generated big datasets can, in turn, be utilized in data-driven computational systems approaches for the characterization of drug–microbiota interactions and, furthermore, to develop approaches that predict drug–microbiota interactions. To this effect, machine learning, a branch of artificial intelligence, is being implemented to utilize large datasets so that drug–microbiota interactions can be predicted. An introduction to machine learning concepts goes beyond the context of this review; however, for those wishing to obtain baseline information on the subject, an excellent overview can be found in the article by McCoubrey et al. [111]. The first predictive model for drug–microbiota interactions was named DrugBug [112]. To develop this model, metabolic enzymes from 491 bacterial genomes were utilized and relevant substrates were identified. These substrates were used to identify structural characteristics and subsequently implement a random forest machine learning model to determine bacterial metabolic enzymes likely to be involved in drug metabolism with an accuracy > 90% [112]. In the seminal study by Zimmerman et al. [105] (Section 5.4) that determined the prevalence of commonly administered drug metabolism by commensal bacteria colonizing the gut, a machine learning approach was implemented to identify structural characteristics of commonly administered oral drugs that increase the probability of bacterial metabolism. In this study, clustering by principal component analysis revealed a collection of functional groups, such as amide and ester groups, in the structure of drugs that increased the probability of bacterial metabolism. Interestingly, functional groups were shown to increase the probability of metabolism by certain bacterial species, such as, for example, amide groups increased the probability of metabolism by Bacteroidetes species [105]. Apart from increasing our understanding of the underlying processes in the bacterial metabolism of drugs, this type of analysis can be advantageous in predicting bacterial metabolism in newly developed drugs.

The issue of compositional changes in gut microbiota induced by administered drugs was addressed by Algavi and Borenstein [113]. The authors developed a machine learning approach to predict such drug–microbiota interactions by incorporating microbial genomic characteristics with regard to biochemical pathways encoded, as well as drug chemical properties. Using the resulting model, interaction predictions were shown to be successful in an in vitro setting but also in in vivo animal model studies as well as in clinical trials. This type of prediction model can present numerous advantages for newly developed drugs and personalized medicine.

Pharmacogenetics utilizes the genomic information of the patient to guide treatment strategies toward maximizing clinical benefits and minimizing risks. Recent advances in genome analysis, such as the DMET^TM^ platform [114] for identification of variants in genes involved in drug assimilation, and next-generation sequencing, have generated enormous amounts of data relevant to genetic characteristics of patients that can be an influencing factor toward successful (or negative) treatment outcomes [115]. As the available data have increased, computational analysis approaches in the context of pharmacogenetics have become more sophisticated and geared toward improving outcome reliability and accuracy. To that effect, due to its flexibility, machine learning has been used in the field of pharmacogenetics where the generated complex data structures accumulated are analyzed with the aim of predicting treatment outcomes [116]. Machine learning approaches have impacted cancer research through their potential for diagnosis and prognosis improvement. Furthermore, machine learning has shown its strength and flexibility by incorporating complex datasets, such as radiology data and tumor genetic profiles (under the coined term radiogenomics), that could potentially be used for non-invasive testing [117]. In the context of cancer treatment, Guo et al. [118] recently implemented machine learning algorithms to predict chemotherapy toxicity, a major barrier to non-adjuvant chemotherapy, in patients with locally advanced cervical cancer by identifying specific single nucleotide polymorphisms associated with toxicity during treatment. In another study, Brindha et al. [119] successfully implemented machine learning algorithms to predict the efficacy of five drugs based on clinical as well as molecular characteristics of oral squamous cell carcinomas. Furthermore, machine learning has recently been implemented in numerous aspects of oncology, such as drug target prediction [120], drug repurposing [121], and prognostic profiles for immunotherapy [122].

In the field of psychiatry, Athreya et al. [123] implemented machine learning algorithms as a predictor of selective serotonin reuptake inhibitor response in patients with major depressive disorder. Six single nucleotide polymorphisms in or near the genes *TSPAN5*, *ERICH3*, *DEFB1*, and *AHR* were included in the method, resulting in a prediction accuracy > 69%. There are numerous studies implementing machine learning predictive models for antidepressant treatment [124,125,126], indicating the drive toward implementing predictive models in this field, as well as their clinical relevance.

Overall, machine learning is gradually developing into a useful tool linking clinical research with clinical practice [127]. As artificial intelligence becomes more sophisticated and more readily available, the integration of complex algorithms in clinical research and practice will become all the more pertinent. The potential benefits could be substantial toward implementing comprehensive patient-tailored precision drug treatments that will maximize the likelihood of benefits based on the genetic and microbiota profile of patients.

## 9. Conclusions

Oral drugs are a popular and patient-friendly option for pharmaceutical treatment strategies. Although oral drugs can be formulated to maximize their bioavailability, there are factors associated with the host that modulate bioavailability and contribute toward drug response interpatient heterogeneity. Some factors are inherently impossible/difficult to overcome, such as the length of GI tract sections and passage time through these sections. Some factors that cannot be changed, such as genetic background, can be factored into patient management so that treatment response or adverse effects can be predicted and potentially avoided, respectively. The microbiota has recently emerged as an important component that interacts with ingested drugs in a complex manner. Understanding the factors influencing drug bioavailability can be used to improve treatment success and avoid adverse effects. Furthermore, the implementation of complex artificial intelligence algorithms integrating the numerous factors affecting drug bioavailability provides the potential for improving personalized treatment strategies in the clinic.

## Data Availability

The data presented in this study are available upon request from the corresponding author.
